# An update on adrenocortical cell lines of human origin

**DOI:** 10.1007/s12020-022-03112-w

**Published:** 2022-06-28

**Authors:** Sandra Sigala, Elisa Rossini, Andrea Abate, Mariangela Tamburello, Stefan R. Bornstein, Constanze Hantel

**Affiliations:** 1 Section of Pharmacology, Department of Molecular and Translational Medicine, University of Brescia, 25124 Brescia, Italy; 2 Department of Endocrinology, Diabetology and Clinical Nutrition, University Hospital Zurich (USZ) and University of Zurich (UZH), 8091 Zürich, Switzerland; 3 Medizinische Klinik und Poliklinik III, University Hospital Carl Gustav Carus Dresden, 01307 Dresden, Germany; 4 Diabetes and Nutritional Sciences, King’s College London, London, WC2R 2LS UK; 5 Center for Regenerative Therapies, Technische Universität Dresden, 01307 Dresden, Germany; 6 Paul-Langerhans-Institute Dresden, Helmholtz Center Munich, University Hospital Carl Gustav Carus, Faculty of Medicine, Technische Universität Dresden, 01307 Dresden, Germany; 7 Lee Kong Chian School of Medicine, Nanyang Technological University, Singapore, 636921 Singapore

## Abstract

Adrenocortical carcinoma (ACC) is a rare, heterogenous and highly malignant disease. Management of ACC is dependent on disease stage with complete surgical resection as the only potentially curative option. However, advanced, un-resectable, metastatic stages and also recurrences often require systemic treatments, which are unfortunately nowadays still unsatisfactory. The scarcity of preclinical models reflecting patient heterogeneities and furthermore drug-resistant phenotypes, has hampered the progress and development of new therapies in recent years. In this review, we provide an overview on the classical models and substantial progress which has been made over the last years in context of this aggressive disease.

Adrenocortical carcinoma (ACC) is a rare and highly malignant disease with an estimated incidence of ∼0.5–2 new cases per million people per year. Clinical treatment of choice is surgery, however, the diagnosis often highly delayed thereby leading to advanced, unresectable and metastatic stages with very limited prognosis and a 5-years overall survival being <15% [1, 2]. Etoposide, cisplatin, doxorubicin and mitotane (EDP-M) constitute the current gold standard for metastatic ACC, which, however is not satisfactory and results frequently in clinical toxicity [1]. The scarcity of human adrenocortical cell lines hampered in recent years more detailed preclinical investigations and the development of strategies considering for example various genotypes, secretion profiles and drug resistant phenotypes. However, since 2016 we observe tremendous progress in this field with overall five new human adrenocortical cell lines developed. In this review, we provide an overview on the current state of the art.

## NCI-H295

**Fig. 1:**
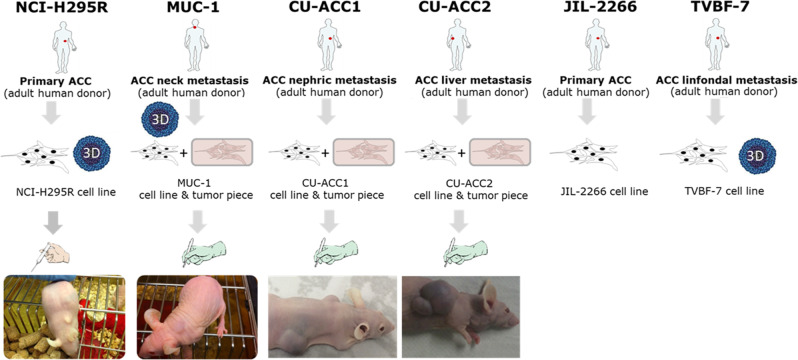
Overview in the currently available human adrenocortical cell lines. Modified from [12]

Gazdar et al. reported in 1990 on the first human adrenocortical cell line, NCI-H295, which was obtained in 1980 from a 48-year-old woman diagnosed with a primary ACC (Fig. 1; [3]). After surgical resection and subsequent processing, the established cells underwent long-term culture over many years. Radioimmunoassays and mass spectrometry revealed then 7 to 9 years after cell line establishment that the cells show the ability to produce all major adrenal steroids including corticosteroids, mineralocorticoids, androgens and estrogens [3]. Since then until today NCI-H295 (including its substrains H295A, H295R, H295RA, HAC13, HAC15 and HAC50) is the most commonly implemented model in context of adrenal steroidogenesis [4–7]. The HAC-substrains result from the attempt to develop a new human adrenocortical carcinoma (HAC) cell line with ACTH responsiveness. Clonal populations of adrenocortical cells were isolated from a tumor of a 11-month-old female patient with hypertension and hirsutism. Later on, single-nucleotide polymorphism analysis revealed that the clones were isolated from contaminated H295R cells. Accordingly, compared to the original NCI-H295 strain, isolated from a mixed population of tumor cells, the HAC subclones are monoclonal [8, 9]. Of note, the reported steroidogenic capacities differ considerably for NCI-H295 and its substrains. This applies also to not specifically selected subclones which naturally occur over time in various labs and are in part maybe also due to varying culture conditions which additionally influence the secretory output [6, 10].

Genetically, NCI-H295 cells are characterized by a large deletion in the TP53 locus [11–13] and are also known to carry an activating CTNNB1 mutation (Table 1, [12–14]). A comprehensive analysis of the mutational landscape of NCI-H295, basing on whole-exome sequencing, is also available since 2019 [15].

**Table 1:** Known mutations of ACC cell lines

Cell line	Mutations	References
**NCI-H295R**	Mutation on **TP53** gene - homozygous deletion of exons 8–9 Mutation on **CTNNB1** gene - c.T133C:p.S45P, activating	[11, 13, 14, 54]
**MUC-1**	Mutation in **TP53** gene - c.1024delC:p.R342fs.	[10, 12, 13].
**CU-ACC1**	Mutation on **CTNNB1** gene - c.G100A:p.G34R, predicted as a gain-of-function	[41]
**CU-ACC2**	Mutation on **TP53** gene - c.G337A:p.G245S, predicted as a loss of function. Mutation on **MSH2** gene - homozygous deletion of exons 1–6	[41]
**JIL-2266**	Hemizygous mutation on **TP53** gene - c.859G > T: p.E287X, stop-gain mutation Mutation on **MUTYH** gene - c.316C > T: p.R106W, loss of function	[48]
**TVBF-7**	Mutation on **APC** gene - c.739C > T:p.Q247*, non-sense	[13]

Gazdar et al. reported in the original study also on NCI-H295’s tumorigenicity when inoculated subcutaneously into athymic nude mice ([3], Fig. 1). Until today NCI-H295 represents, thus, also the most commonly used subcutaneous xenograft model for human ACC and was later also applied as ACC hepatic metastasis model [16, 17]. Accordingly, various preclinical studies have been performed since 1990 in pathophysiological and therapeutic context. It could be for example demonstrated, that Wnt/β-catenin pathway [12, 13] and SF-1 modulations are involved in pathogenesis and potential targets for the treatment of ACC [18, 19]. NCI-H295 were also repeatedly implemented in context of IGF-System and IGF1-receptor inhibiting approaches in ACC [20–23]. Using NCI-H295 as a tool, it could be furthermore demonstrated that mitotane is an inhibitor of sterol-O-acyl-transferase 1 (SOAT1) leading to accumulation of toxic lipids and subsequent ER stress [24]. Studies including NCI-H295 enabled also the investigation of preclinical experiments investigating the current clinical gold standard EDP-M (etoposide, doxorubicin, cisplatin, mitotane) and potential next-generation schemes involving nano-technological modified variants of the parental drugs [25, 26].

Nowadays, NCI-H295 is commonly implemented together with the next-generation models outlined below. Thus, further examples and most recent pre-clinical implementation of NCI-H295 will be context-dependent added in the following sections.

## MUC-1

In 2016, we have reported on MUC-1, the second human adrenocortical cell line developed in this field. In contrast to the chemo-naïve and often pre-clinically good responding NCI-H295, MUC-1 represents a clinically systemically treated, but EDP-M-resistant model obtained from a distant ACC metastasis of a male patient. MUC-1 has been established as cell line for in vitro, but also as patient-derived tissue xenograft for in vivo settings (Fig. 1, [10, 23]). The model shows the for metastases frequently observed increased cellular plasticity, which can be e.g. seen in highly elevated drug tolerance for a wide range of chemotherapeutic, phytochemical, molecular targeted drugs and also combinatory regimens which is far beyond resistance against the originally applied EDP-M scheme [10, 27–36]. Recently, a broad comparative drug screen of clinically relevant chemotherapies and targeted therapies has been performed in NCI-H295R and MUC-1. Interestingly, these experiments identified the very classical combination gemcitabine and cisplatin as highly promising treatment, but indicated in parallel also strong molecular signs of acquiring gemcitabine resistance as represented by treatment-dependent ribonucleotide reductase-upregulation in both tumor models. Of note, a combination of gemcitabine, cisplatin and the dual ribonucleotide-reductase-inhibitor COH-29 led to previously total cell killing for both tumor models [33].

Also regarding metabolic re-programming MUC-1 provides new insights and opportunities. Warde et al. recently initiated comprehensive analyses in terms of lipid metabolism and found significant differences in cholesterol storage and lipid droplet re-modelling between mitotane sensitive (NCI-H295R) and resistant (MUC-1) ACC cells [31, 37]. Fittingly, we found recently also signs of differential HSD17B4 regulation in MUC-1, a gene with a dual role in steroidogenesis and fatty acid oxidation [13]. HSD17B4 expression is also known to be involved in the development of castration resistant prostate cancer, leading to metabolic re-programming and poor prognosis [38, 39]. In this context we have lately also characterized steroidogenic signaling in a panel of ACC cells [13]. In accordance with the original patient record of a diffuse steroidogenic phenotype, MUC-1 demonstrates here basally a profile with comparably low steroidogenic activity and secretion. However, e.g. upon forskolin stimulation MUC-1 shows tremendous steroidogenic activity for all steroidogenic precursors, strong electrophysiological response and specific focus on the androgen axis in the resulting functional secretion profile. While MUC-1 demonstrates under these conditions furthermore specific upregulation of the androgen receptor gene, converse regulations are detectable for NCI-H295R. While even a role of adrenal derived androgens is under discussion in castration resistant prostate cancer [40], comprehensive preclinical investigations in terms of androgenic signaling in ACC are currently widely lacking. Basing on our newest findings, MUC-1 represents in this context the most promising cell line.

Moreover, as recently reported for the first time at the 8th International ACC Symposium in Brescia, Italy 2021, further experiments indicate for MUC-1 spontaneous metastatic potential which could be not reported for any other human ACC cell line so far (https://www.acc2021brescia.com/). Recently published experiments on the chemokine axis and related tumor aggressiveness confirm differential regulations for CXCR4 and CXCL12 in NCI-H295R and MUC-1, which also suggest varying potential in terms of metastasization [34].

Genetic analyses demonstrate for MUC-1 a somatic deletion/frameshift mutation in TP53 (Table 1), but no mutations for example in MEN1, PRKAR1A, CTNNB1, APC, ZNRF-3, IGF-2, EGFR, RB1, BRCA1, BRCA2, RET, GNAS and PTEN [10, 13]. Whole genome and whole-bisulfite sequencings have been performed for the panel of NCI-H295, MUC-1 and a new ACC cell line named TVBF-7 (please see below). These overall data sets will be available soon.

## CU-ACC1 and CU-ACC2

In 2018, two further cell lines were established from metastatic ACC in female patients, namely CU-ACC1 and CU-ACC2 (Fig. 1, [41]). The CU-ACC1 cell line derives from a perinephric ACC metastasis [41] and secretes higher levels of cortisol compared to NCI-H295R cells, while no aldosterone secretion was reported. Genetic analysis reveals an activating point mutation of CTNNB1 gene [41], a well known ACC driver gene [42]. The CU-ACC2 cell line was established from a liver metastasis in a Lynch syndrome patient with non-secreting primary ACC [41]. Despite high abundance of CYP11A1 [43], CU-ACC2 cells secrete only minute amount of cortisol and are unresponsive to hormone stimulation [41]. CU-ACC2 cells represent wild type for CTNNB1, while a mutation in TP53 is reported (Table 1, [7, 41]). In addition, in line with the Lynch syndrome, a heterozygous deletion of 1–6 exons of MSH2 gene was detected. The mutation on the CTNNB1 gene found in CU-ACC1 is within the ubiquitination recognition motif. Currently, this mutation is not fully characterized but it is predicted to be activating [41]. These ACC experimental cell models, together with the NCI-H295R cells, have been recently used to demonstrate sensitivity of ACC to ferroptosis and its dependence on the active steroid synthetic pathways [43]. Moreover, these models were useful to identify the maternal embryonic leucine zipper kinase (MELK) [44], the mitotic PDZ-binding kinase (PBK) [45] and inhibition of aurora kinase and beta-catenin pathway as potentially druggable targets for ACC [46]. Furthermore, the CU-ACC2 cell line allowed to set up the first humanized ACC patient-derived xenograft mouse [47], elucidating the effects of immunotherapy and its mechanism of action. Authors suggested that the effects of an anti-PD1 on tumor microenvironment would recapitulate effects of the immune milieu of the tumor in the matching patient.

## JIL-2266

JIL-2266 is a newly established ACC cell line presenting high tumor mutational burden (TMB) and loss of heterozygosity (LOH) [48]. Similar to NCI-H295R cells, JIL-2266 is derived from a female patient with a primary ACC (Fig. 1). The subsequent characterization of JIL-2266 revealed intermediate-to-low expression of SF-1, while the hormonal production and steroidogenic enzyme expression is reported to depend on culture media composition [48]. Exome sequencing demonstrates the presence of a pathogenic germline mutation in the MUTYH gene, which is linked to base excision repair inactivation, leading to high TMB. Further genetic analysis showed a hemizygous stop-gain mutation in the TP53 gene (Table 1), which presented high expression, and somatic nucleotide variant in the ZNRF3 gene. Similar to MUC-1 and CU-ACC2, JIL-2266 holds wild type CTNNB1.

## TVBF-7

Recently, the new ACC cell line TVBF-7 has been proposed by our group [13]. These cells represent the most recently developed ACC cell line, formerly known as primary cell culture ACC115m [35, 36]. Cells were established from a perirenal lymph-node metastasis of a male ACC patient which, similar to MUC-1, underwent progression upon clinical EDP-M treatment [13]. After establishment of a primary cell culture, the cells were found to be continuously passageable. Moreover, STR-analysis revealed a unique STR-profile, which could be furthermore linked to the original patient tumor [13]. Interestingly, although the patient did not report signs of hormone excess, TVBF-7 cells demonstrate basally high levels of cortisol and related candidates of the glucocorticoid axis. Moreover, they are unresponsive to a selection of known physiological stimuli, thereby indicating autonomous cortisol secretion [13, 49]. Fittingly, further genetic investigations indicate an altered Wnt/β-Catenin pathway due to the detection of a non-sense APC mutation (Table 1). Except for the ACTH receptor (MC2R), these cells express, compared to NCI-H295R, low level of hormonal receptor at gene level [13, 35]. The expression of estrogen receptor α is comparable to that of NCI-H295R, while the expression of estrogen receptor β is lower [35]. TVBF-7 cells have already been used as an experimental cell model to test the efficacy of drugs proven effective in ACC, in addition to other ACC cell lines. Indeed, we demonstrated that progesterone receptors may be a druggable target in these ACC cells [35, 36], thus confirming the correlation between the level of the progesterone receptor expression and the sensitivity of ACC cells to the cytotoxic effect elicited by progesterone [35, 36, 50, 51]. Finally, the Cdk4/6 inhibitor ribociclib induces cytotoxicity and reduction of the cell proliferation rate in these cells, although the cell cycle perturbation appears to differ compared to other ACC cell lines. TVBF-7 cells accumulate, as expected, in G1 phase, while NCI-H295R cells and MUC-1 cells accumulate in G2 phase [36], thus supporting the experimental advantage to have different cell models that could reflect the different ACC behavior, both in vitro and in vivo. Interestingly, in line with the shared metastatic origin from patients in progression after EDP-M, the TVBF-7 cells reflect some, but not all, characteristics with the cell line MUC-1. In a recent published work, the exposure of these both cell models to mitotane, to progesterone and to the CDK4/6 inhibitor ribociclib alone or in combined setting, revealed a lower sensitivity compared to the NCI-H295R cells [36]. The different responses to pharmacological treatments underline the importance of their different phenotype.

Finally, a detailed report on the establishment of ACC tumor spheroids and organoids including NCI-H295R, MUC-1 and TVBF-7 in a high-throughput format is currently under review. A pre-print of the manuscript can be found here (Fig. 1, [52]).

## Conclusions

In sum, a lot of progress has been made over the last years and changed the preclinical landscape for ACC. The new ACC experimental cell models, together with the worldwide-used NCI-H295R cell line, give to researchers instruments that are in line with the well known heterogeneity of this disease and have the potential to reveal so far unknown patient sub-type characteristics. Furthermore, ACC cell lines of metastatic origin can offer useful models to investigate the pharmacological effect of drugs and the pathological molecular alteration in the context of metastatic and EDP-M-progressed disease, which is specifically challenging in the clinic management and closely related with poor prognosis. Finally, we would like to underline a key point in using cancer cell lines in research and drug development, that is the risk of cell line misidentification [53]. A periodically authentication by STR analysis as well as the expression of ACC markers, such as Steroidogenic Factor 1 could help to avoid this.
